# Fabrication of Superhydrophobic Magnetic Sawdust as Effective and Recyclable Oil Sorbents

**DOI:** 10.3390/ma12203432

**Published:** 2019-10-20

**Authors:** Shumin Fan, Shuai Pei, Tianyu Shen, Guangri Xu, Yuanchao Li, Wenxiu Fan

**Affiliations:** School of Chemistry and Chemical Engineering, Henan Institute of Science and Technology, Xinxiang 453003, Henan, Chinaxugr70@163.com (G.X.); liyuanchaozzu@126.com (Y.L.)

**Keywords:** sawdust, superhydrophobic, magnetic, oil absorption, emulsion separation

## Abstract

In this paper, a novel superhydrophobic magnetic sawdust (SMSD) was fabricated as an oil sorbent. The SMSD was functionalized with Fe_3_O_4_ nanoparticles using melamine formaldehyde resin (MFR) as a coupling agent and subsequently hydrophobically-treated with hexadecyltrimethoxysilane (HDTMS). The SMSD showed excellent superhydrophobicity with the water contact angle of 155.3 ± 0.9°. Meanwhile it had remarkable environmental durability, long-term stability, and mechanical durable properties. Taking advantage of its magnetic characteristics, the SMSD could be easily controlled to absorb oil to separate oil–water mixtures with high oil absorption capacity and good reusability. Moreover, the emulsion was successfully separated by SMSD, including water-in-oil and oil-in-water emulsions. This study developed an effective oil absorbent, which was low cost and environmentally-friendly.

## 1. Introduction

Oil spill problems have become more and more serious in water pollution, which is a major threat to human life and the ecosystem [1,2,3,4]. Therefore, more and more attention has been paid to efficient absorbent materials for oil spill pollution, such as porous materials, activated carbon, chemical synthetic organic materials, and oil absorption resin [5]. However, these absorbents inevitably encounter some drawbacks, including being time-consuming, high-priced, and non-biodegradable, having low separation efficiency, secondary pollution, and the difficulty in collecting, co-absorption of water, et al. [6]. For this reason, biodegradable natural materials with superhydrophobicity and high separating capacity are urgently needed.

In recent years, superhydrophobic surfaces have been focused with the apparent contact angle of water (WCA) larger than 150° and sliding angle (SA) lower than 10° [7]. The superhydrophobicity could be generally attributed to the combination of geometric microstructure and surface chemical composition [8]. It could also be created through surface microstructure modification without a low surface-energy coating [9,10]. The most widely used technologies for preparing superhydrophobic materials include the template method [11], chemical etching [12], chemical or physical vapor deposition [13], layer-by-layer self-assembly [14], thermal decomposition [15], electrostatic spinning [16], the sol–gel process, and impregnation technology [17]. With these methods, various superhydrophobic surfaces have been manufactured by using metals [18,19], polymers [20,21], glass [22,23], fiber glass [24], paper [25,26], corn straw [7], and sawdust [27]. Zang et al. [27] adopted a self-assembly method to achieve a superhydrophobic and superoleophilic surface by deposition of SiO_2_ particles on a fiber surface and self-assembled octadecyltrichlorosilane, which successfully removed various oils from water. Sun et al. [28] fabricated a hydrophilic wood surface using the hydrothermal method by coating ZnO nanorod arrays on the wood surface and modification of n-dodecyltrimethoxysilane. For the effect of surface roughness and lower surface energy, the wood product exhibited superhydrophobicity with the WCA of 156°. As a renewable natural resource, sawdust is a readily available by-product of the wood and paper industries. Compared with the traditional adsorbent, sawdust is an effective substrate to prepare a superhydrophobic surface for oil absorption. However, to the best of our knowledge, there are relatively few reports about sawdust as a superhydrophobic magnetic oil absorbent material.

In this study, sawdust was selected as the substrate to prepare a superhydrophobic oil sorbent by the coating of Fe_3_O_4_ nanoparticles and self-assembly of hexadecyltrimethoxysilane (HDTMS) monomer with melamine formaldehyde resin (MFR) as a coupling agent. The viscidity of MFR enhanced the superhydrophobicity of the product. Meanwhile, the sawdust coated with Fe_3_O_4_ nanoparticles could be recycled by magnetic force. The superhydrophobic magnetic sawdust (SMSD) showed excellent oil absorption capacity from an oil/water mixture. In addition, the SMSD successfully separated oils from water-in-oil and oil-in-water emulsions. 

## 2. Experimental

### 2.1. Materials

Melamine (C_3_H_6_N_6_), methenamine (C_6_H_12_N_4_), formaldehyde (HCHO), triethanolamine ((HOCH_2_CH_2_)_3_N), HCl, NaOH, HAc, NaAc, FeCl_2_, FeCl_3_, NH_3_·H_2_O, NH_4_Cl, anhydrous ethanol (CH_3_CH_2_OH), hexadecyltrimethoxysilane (HDTMS, C_19_H_42_SiO_3_), Sudan III, ethyl acetate (C_6_H_10_O_3_), and acetic acid (CH_3_COOH) were purchased from Shanghai (China) Aladdin Biochemical Technology Co. Ltd. All chemicals were of analytical grade and were used without further purification. All solutions were made with deionized water. The deionized water used throughout this research was self-made. The sawdust was purchased from the local market, and it was sifted through 40 and 60 mesh screens to obtain a uniform size between 250 μm and 425 μm. 

### 2.2. Methods

#### 2.2.1. Preparation of Melamine Formaldehyde Resin (MFR)

Methenamine (0.125 g) dissolved in formaldehyde solution was mixed with 31.5 g of melamine stirring at 75 °C in a water bath for 1 h with a condensing reflux device. Afterwards, the precipitation ratio was measured. When the precipitation ratio reached 2:2, 0.15 mL of triethanolamine solution was added. For this, 2 mL of solution was taken out. After cooling down to 20 °C, 2 mL of deionized water was added dropwise. When the solution became slightly turbid, the precipitation ratio was 2:2. After a few minutes, the MFR was obtained. The reaction was as follows:
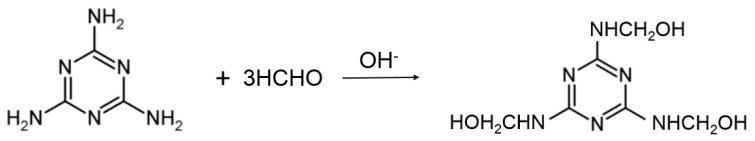


#### 2.2.2. Preparation of Magnetic Sawdust

Fe_3_O_4_ nanoparticles was prepared by the water co-precipitation method. The aqueous solution containing FeCl_2_ and FeCl_3_ was deoxygenated by N_2_ and heated to 50 °C. Then, NH_3_·H_2_O was rapidly added. After the reaction, the black magnetic particles were attracted by magnet, which were washed with deoxygenated water three times for use. The resulting product was dried at 60 °C for 2 h in a vacuum oven (Shanghai feiyue experimental instrument Co. LTD, Shanghai, China). The powder obtained was Fe_3_O_4_ nanoparticles.

Sawdust (0.5 g) was immersed into 15% MFR solution for 15 min at 70 °C in a water bath. Subsequently, the sawdust was separated out through filtration. The MFR coated sawdust was mixed with Fe_3_O_4_ powder. The mixture was kept in the oven for 30 min at 50 °C. Then, the product was immersed into 15% MFR solution again. The Fe_3_O_4_ nanoparticles were wrapped in viscid MFR by a structure of MFR–Fe_3_O_4_ nanoparticles –MFR. Thus, Fe_3_O_4_ nanoparticles could tightly adhere to the sawdust. 

#### 2.2.3. Modification of Magnetic Sawdust

**Scheme 1:**
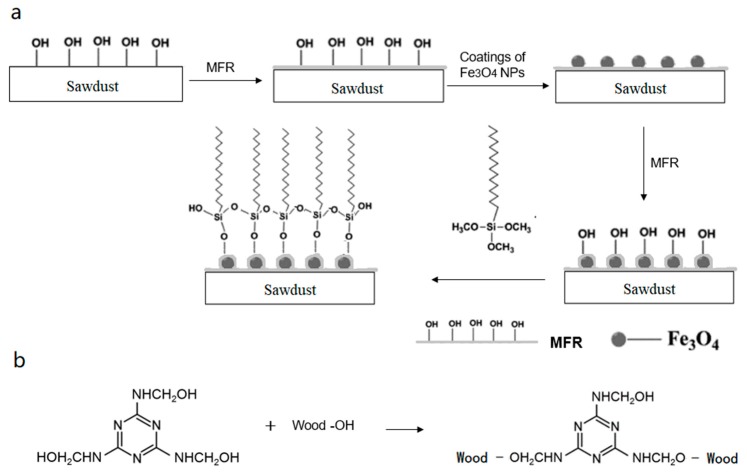
(**a**) Illustration for the preparation procedure of superhydrophobic magnetic sawdust (SMSD); (**b**) the reaction between sawdust fiber surface and melamine formaldehyde resin (MFR).

The modification of the magnetic sawdust was performed by dehydration condensation reaction between HDTMS and the MFR layer on sawdust. HDTMS (2 mL) was mixed with 100 mL anhydrous ethanol, 0.25 mL deionized water, and 0.05 mL acetic acid to form HDTMS–ethanol solution. The magnetic sawdust was immersed into the HDTMS–ethanol solution at 60 °C for 4 h. After drying, the superhydrophobic magnetic sawdust was successfully obtained, which was abbreviated to SMSD. Scheme 1 shows the illustration for the preparation of SMSD.

### 2.3. Characterization

The chemical composition of sawdust samples was analyzed using Fourier transform infrared spectroscopy (FT-IR, Magna-IR 560) (Thermo Nicolet, Madison, WI, USA), X-ray diffraction (XRD, D8 ADVANCE) (Bruker AXS, Karlsruhe, Germany) and X-ray photoelectron spectroscopy (XPS, Thermo Scientific Escalab EI+) (Thermo Scientific, Waltham, MA, USA). The surface morphology of the samples was characterized with a scanning electron microscope (SEM, Quanta 200) (FEI, Hillsboro, OR, USA). The apparent contact angle of water (WCA) and the apparent contact angle of oil (OCA) values were measured at ambient temperature with an optical contact angle meter (Shenzhen testing equipment CO., LTD, Shenzhen, China, TST-200H). The WCA was measured by dropping 5 μL of deionized water onto the compacted surface of sawdust. The OCA was measured using diesel oil. The WCA values were obtained from the average of three measurements.

### 2.4. Oil Absorption Performance

The oil absorption performance was evaluated by maximum oil absorption capacity. The maximum oil absorption capacity was measured in a pure oil system. A nylon net bag filled with 0.5 g of sawdust was dipped into a beaker containing 150 mL of oil at room temperature. After 5 min, the nylon net bag was removed with a nipper. The oil-saturated sawdust was taken out from the nylon net bag and weighed. The maximum oil absorption capacity was calculated by:

(1) $$q=\left(m_{2}-m_{1}\right)/m_{1},$$

where q is the maximum oil absorption capacity (g g^−1^); *m*_1_ is the initial weight of sawdust before absorption (g); *m*_2_ is the weight of sawdust after absorption (g).

Then, the oil-saturated sawdust was centrifuged for 5 min to remove the oil to obtain a regenerated product. The process of oil absorption–oil release is one cycle. After being rinsed in acetone and water, the regenerated product could be reused for many cycles.

## 3. Results and Discussion

### 3.1. Composition and Morphology

**Figure 1:**
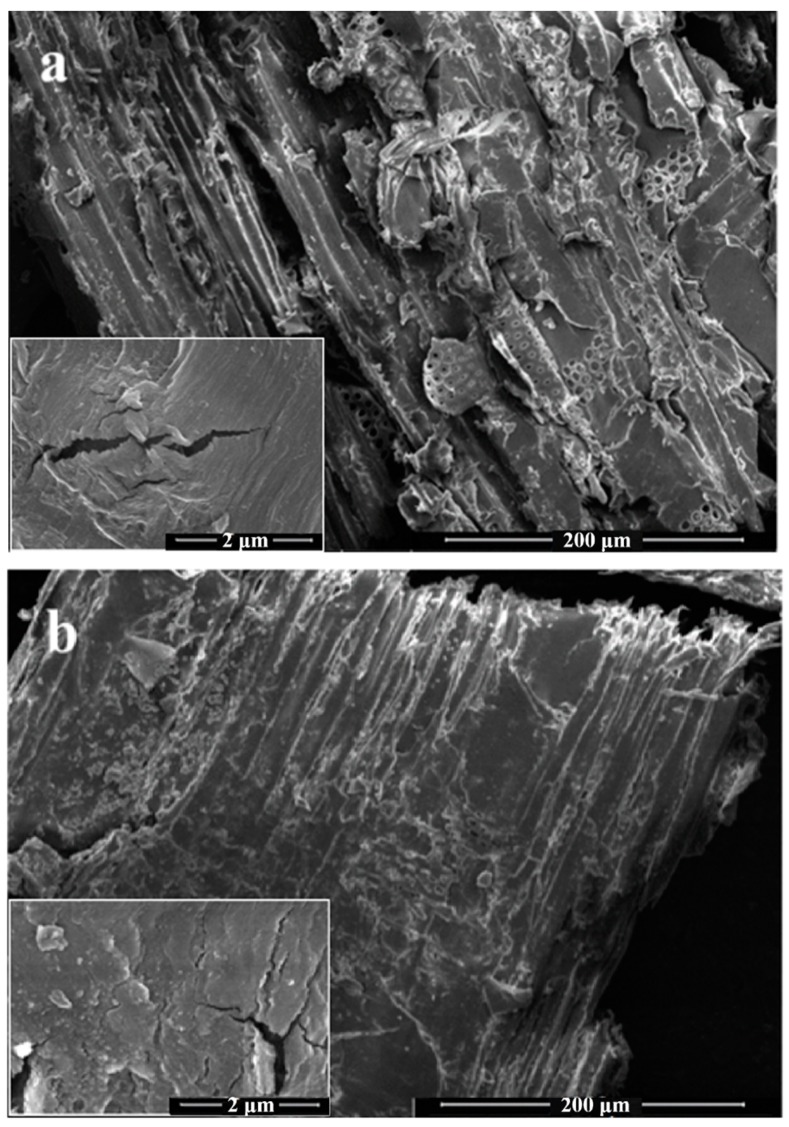
SEM images of raw sawdust (**a**) and SMSD (**b**).

The surface chemical components and morphology of raw sawdust and SMSD were detected by SEM, FT-IR, XRD, and XPS. Figure 1 shows the SEM images of raw sawdust and SMSD. The SMSD was different from the raw sawdust in surface morphology. It could be seen that the external surface of SMSD was covered with a membrane. This was due to the coating of the transparent MFR layer on the sawdust. Thus, the fiber structure of sawdust could still be observed. The samples were also observed at high magnifications. Compared with raw sawdust, the Fe_3_O_4_ nanoparticles were observed on the surface of SMSD. 

**Figure 2:**
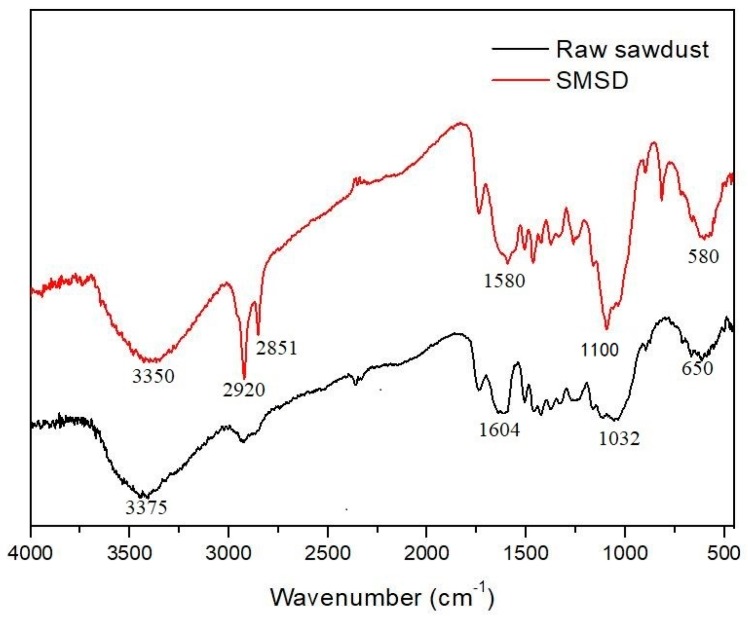
FTIR spectra of raw sawdust and SMSD.

The typical FT-IR spectra of sawdust over the range of 500–4000 cm^−1^ is presented in Figure 2. The peaks at 3300–3500 cm^−1^ in both spectra were for the bonded –OH stretching vibrations. The asymmetric and symmetric stretching vibrations of –CH_2_ and –CH_3_ were at 2920 cm^−1^ and 2851 cm^−1^, confirming the existence of HDTMS. Moreover, the small absorption peaks around 1100 cm^−1^ and 800 cm^−1^ observed in SMSD corresponded to the Si–O–Si and C–Si stretching vibration of HDTMS, which was overlapped by the C–O stretching vibration of the sawdust. The absorption peak at around 580 cm^−1^ in SMSD corresponded to the characteristic absorption peak of Fe_3_O_4_. The FT-IR analysis confirmed that the functionalized sawdust was successfully synthesized.

**Figure 3:**
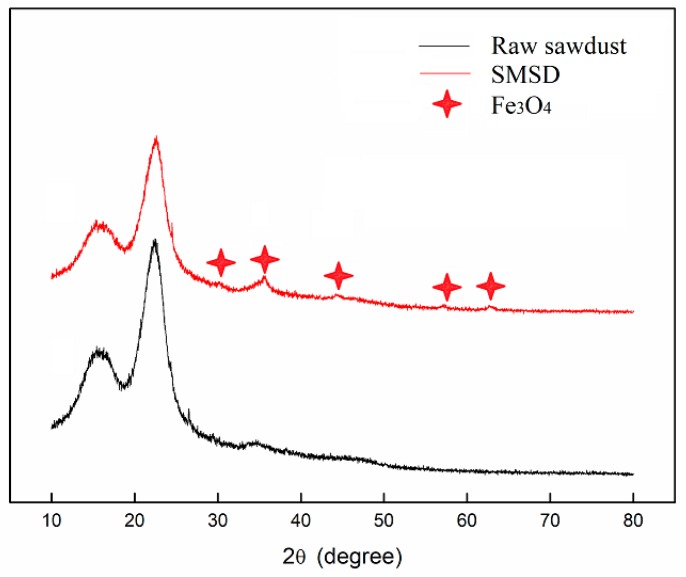
XRD spectra of raw sawdust and SMSD.

The XRD spectra of the samples is shown in Figure 3. The raw sawdust had typical diffraction peaks at 2θ = 15.4°, 22.5°, 26.2°, and 33.9°, and the typical diffraction pattern still existed in SMSD. The diffraction peaks at 30°, 35.3°, 42.9°, 57°, and 62.4°, which corresponded to the (220), (311), (400), (511), and (440) lattice planes of the cubic phase of Fe_3_O_4_, was observed in SMSD. The XRD analysis suggested that the function of Fe_3_O_4_ and HDTMS had little effect on the crystallization property of sawdust fabrics. 

**Figure 4:**
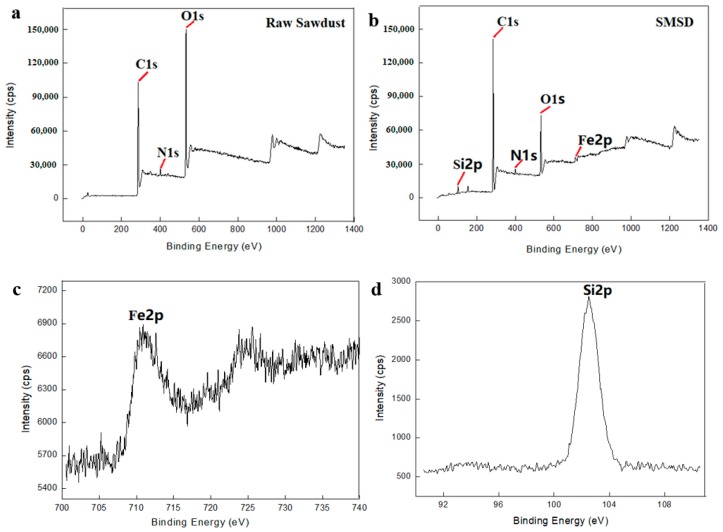
X-ray photoelectron spectroscopy (XPS) survey spectra of raw sawdust (**a**), SMSD (**b**); the high-resolution XPS spectra of Fe 2p (**c**) peaks and Si 2p (**d**).

The XPS analysis was conducted to study the surface composition and bonding environment. Figure 4 showed the XPS of raw sawdust and SMSD. The carbon (C) peak and the oxygen (O) peak streamed from raw sawdust was observed in Figure 4a. In Figure 4b, the new characteristic peaks with binding energy of 710.7 and 102.5 corresponded to Fe 2p (Figure 4c) and Si 2p (Figure 4d). The weak peak of Fe (2p) suggested the thin layer deposition of Fe_3_O_4_ nanoparticles. The Fe_3_O_4_ nanoparticles were chemically covered on the sawdust surface to construct the magnetic superhydrophobic sawdust.

### 3.2. Surface Wettability

**Figure 5:**
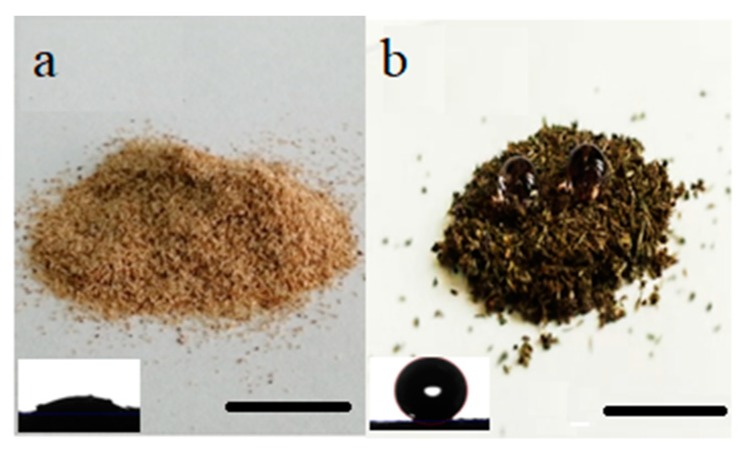
Photographs of (**a**) raw sawdust; (**b**) SMSD. Scale bar, 1 cm.

Figure 5 shows the photographs of raw sawdust and SMSD. The color of SMSD became dark brown due to the modification of brown Fe_3_O_4_ nanoparticles. When water was dropped onto the raw sawdust, the water was absorbed immediately with a WCA of 0° due to the abundant hydroxyl groups on its fiber surface, while the droplet stayed spherical and stable on the surface of SMSD with a WCA of 155.3°. The sawdust covered with MFR and subsequently modified with HDTMS was fabricated as comparison, which was abbreviated to MHSD. The MHSD displayed hydrophobicity with a WCA value of 134.1°. The modification of HDTMS increased the hydrophobicity of sawdust. The SMSD with modification of the Fe_3_O_4_ nanoparticles and self-assembly of HDTMS had a WCA of 155.3°, showing the superhydrophobic characteristics. In addition to the magnetic characteristics, the modification of Fe_3_O_4_ nanoparticles might have helped to increase the surface roughness and lower its surface energy of sawdust, leading to an enhancement of hydrophobicity.

**Figure 6:**
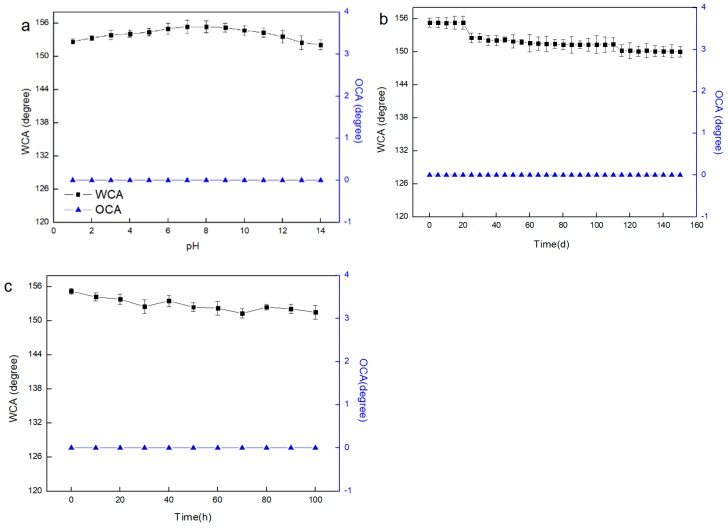
Change of contact angle of water (WCA) and oil (OCA) of SMSD under (**a**) different pH of aqueous solution; (**b**) long-term exposure to ambient air; (**c**) 35 °C and relative humidity of 90% condition.

The chemical stability and environmental durability were investigated under harsh conditions, which had significant effects on practical use. Changes in WCA and OCA of SMSD were measured by changing the pH of the aqueous solution from 1 to 14 for evaluation of chemical stability. The different aqueous solutions with pH varying from 1 to 14 were prepared using a HCl solution, HAc– NaAc buffer solution, deionized water, NH_3_–NH_4_Cl buffer solution and NaOH solution. The SMSD was kept in the aqueous solutions with different pH for 0.5 h. Then, the WCA and OCA were determined. The results were shown in Figure 6a. The WCA values varied slightly with all values higher than 150°, and the OCA values remained 0°. Therefore, the product could keep its superhydrophobic and superoleophilic properties even in corrosive solutions, showing admirable chemical stability. The environmental durability was measured by exposing the SMSD to ambient air for 150 days. As shown in Figure 6b, the WCA decreased slightly from 155.3° to 150.6° after 150 days without significant changes, indicating remarkable environmental durability under atmospheric conditions. Water vapor is very small therefore it could penetrate the pores of sawdust and attack the superhydrophobic surface. Thus, the durability of superhydrophobic materials to water vapor were evaluated. The SMSD was subjected to 35 °C and relative humidity of 90% for 100 h in a constant humidity box. Figure 6c showed the results of WCA and OCA in moisture. The variation of WCA was very small, confirming long-term durability in humidity environments. Hence, the SMSD exhibited long-term durability under harsh conditions, which could be applicable in different environments. 

**Figure 7:**
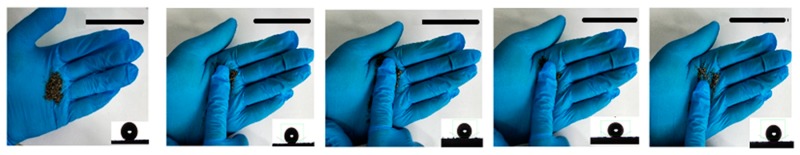
The finger wiping test after 0 (WCA: 155.3°), 20 (WCA: 155.4°), 40 (WCA: 154.9°), 60 (WCA: 153.0°), 80 (WCA: 151.0°) cycles. Scale bar, 5 cm.

To investigate the mechanical stability of the superhydrophobic surfaces of SMSD, the superhydrophobic surfaces were tested after finger wiping and 80 cycles were performed. A pressure was applied onto the surface of SMSD forth and back, which was defined as one abrasion cycle. As shown in Figure 7, the SMSD after abrasion retained the superhydrophobic features with the WCA greater than 150°, indicating excellent mechanical robustness. The good mechanical stability against abrasion could be attributed to the adequate firm combination of the superhydrophobic coating.

### 3.3. Application

#### 3.3.1. Oil Absorption

**Figure 8:**
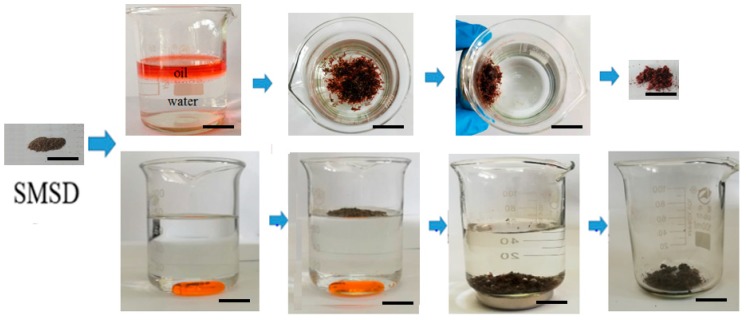
Photographs of absorption of ethyl acetate and tetrachloromethane (colored with Sudan III) with SMSD. Scale bar, 2 cm.

The superhydrophobicity of SMSD provided strong potential for its application in oil spill cleanup. The absorption experiment was designed to test the oil absorption capacity of SMSD for oils with different densities. Figure 8 shows the process of SMSD as an oil sorbent for removal of ethyl acetate and tetrachloromethane (colored with Sudan III) from water. The density of ethyl acetate is less than water, therefore it floats on the surface of water. On the contrary, tetrachloromethane sinks into water due to the greater density than water. The SMSD was thrown into the mixture of water and oil, and the sorption time was 5 min. When the SMSD was dipped into the mixture of water and ethyl acetate, the ethyl acetate was absorbed quickly. The high buoyancy of sawdust meant it floated on the water surface even after absorption. The SMSD could be collected by a magnet, which was crucial for its recycling. When the SMSD was dipped into the mixture of water and tetrachloromethane, the floating SMSD could be attracted to the bottom by a magnet, absorbing the tetrachloromethane quickly. The SMSD filled with oil could be separated from water by a magnet. Thus, the magnetic characteristics made SMSD an excellent oil absorbent for oils with different densities and easy for collection and recycling.

**Figure 9:**
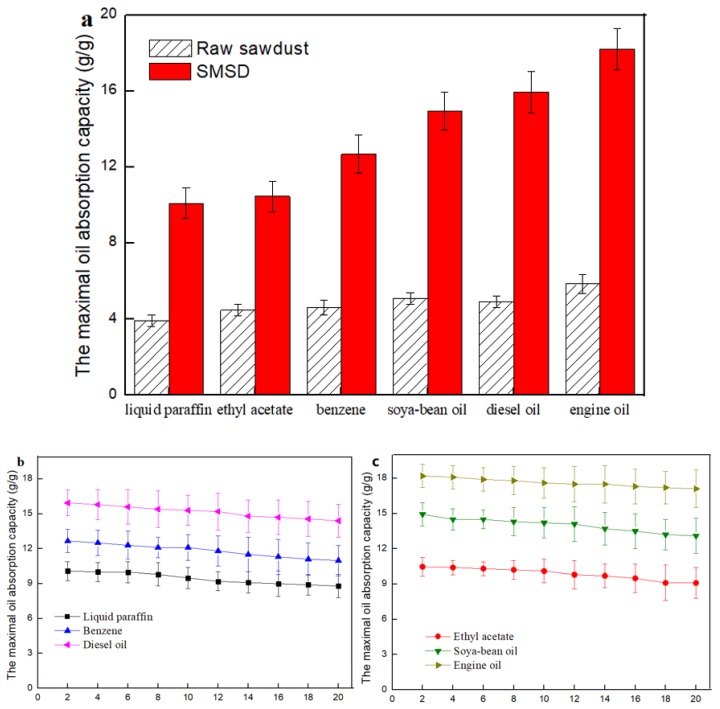
The maximum oil absorption capacity with SMSD: (**a**) for different oils in the first cycle; (**b**,**c**) after different cycles.

The maximum oil absorption capacity for liquid paraffin, ethyl acetate, benzene, soya-bean oil, diesel oil, and engine oil was analyzed. The surface tension values of different oils were shown in Table 1. In Figure 9a, the maximum oil absorption capacity of SMSD was almost three times that of raw sawdust for different oils, indicating the significant improvement of oil absorption capacity. The interaction between sawdust and oils is physical absorption, which is caused by molecular force. The absorption capacity of physical absorption depends on the similarity of polarity between absorbent and solution, leading to its low selectivity. Thus, the oil absorption performance of SMSD varied with the oils. The reusability of the SMSD was investigated by determination of maximum oil absorption capacity for 20 cycles, which is presented in Figure 9b. After 20 cycles, the SMSD still maintained high maximum oil absorption capacity, with a slight decline, indicating its stability of oil absorption.

#### 3.3.2. Emulsion Separation

**Figure 10:**
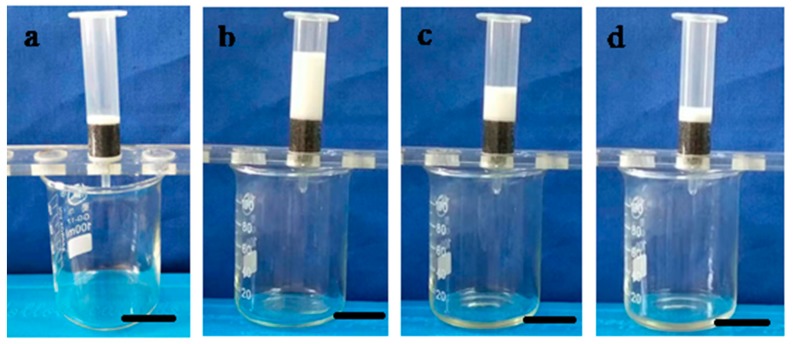
(**a**) The designed apparatus for separation of water-in-oil emulsion using SMSD; (**b–d**) the filtration process for separation of water-in-tetrachloromethane emulsion using SMSD. Scale bar, 3 cm.

**Figure 11:**
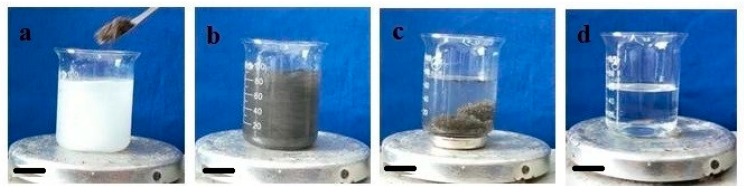
The separation of oil-in-water emulsion using SMSD: (**a**) adding of SMSD; (**b**) after 1 min; (**c**) after 5 min; (**d**) the separated water. Scale bar, 2 cm.

The separation capacity of SMSD for emulsion was also investigated. The surfactant-stabilized water-in-oil and oil-in-water emulsions were prepared. The water-in-oil emulsion was prepared through mixing of the surfactant (Span 60 0.1 wt%), water (1 wt%), and oil (tetrachloromethane, 98.9 wt%) under high stirring. The oil-in-water emulsion was prepared through mixing oil (tetrachloromethane, 1 wt%) and water (98.9 wt%) with the addition of surfactant (sodium dodecyl sulfate 0.1 wt%). Before separation, the emulsion was a milky white liquid. A solid phase extraction column was used for separation of the water-in-oil emulsion. The SMSD was placed in the column with the upper and the lower ends of a supporting plate, which is shown in Figure 10a. Because of the superhydrophobic and superoleophilic characteristics of SMSD, the tetrachloromethane was absorbed in SMSD and flowed out from the bottom, which was transparent compared to the feed. Thus, the water-in-tetrachloromethane emulsion was successfully separated by SMSD. In Figure 11a–d, the oil-in-water emulsion was separated by SMSD under stirring for 5 min. As shown in Figure 11a, the color of the oil-in-water emulsion was milky white. Then, the SMSD was added into the emulsion. The color of emulsion became dark in 1 min (Figure 11b). After 5 min, the stirring was stopped while a magnet was put under the beaker. The SMSD was attracted to the bottom (Figure 11c). Taking advantage of the magnetic characteristics of SMSD, the water could be separated out. After filtration, the purified water was obtained (Figure 11d). In summary, the SMSD could successfully separate the oil from water-in-oil emulsion and oil-in-water emulsion. The separation process for surfactant-stabilized emulsion is shown in Videos S1 and S2.

## 4. Conclusions

In this study, a novel superhydrophobic magnetic sawdust was successfully fabricated via the coating of Fe_3_O_4_ wrapped by sticky MFR and self-assembly of HDTMS. The wettability tests exhibited that the SMSD had a WCA of 155.3° and oil contact angle of 0°. Meanwhile it had remarkable chemical stability and environmental durability, even in a moisture-rich condition. In addition to the magnetic characteristics, the modification of Fe_3_O_4_ may have helped to increase the surface roughness, leading to an enhancement of hydrophobicity. The SMSD showed excellent oil absorption capacity for different oils from water-oil mixtures. In addition, the emulsion (including water-in-oil and oil-in-water emulsion) was successfully separated by SMSD, providing new insight to environmentally-friendly sawdust as promising sorbents for spilled oil.

## Figures and Tables

**Table 1:** The surface tension values of oils.

Oils	Liquid Paraffin	Ethyl Acetate	Benzene	Soya-Bean Oil	Diesel Oil	Engine Oil
Surface tension (mN·m^−1^)	26.2 (60 °C)	26.3 (25 °C)	28.2 (25 °C)	34.2 (25 °C)	26.8 (25 °C)	40.5 (25 °C)

## Supplementary Materials

The following are available online at https://www.mdpi.com/1996-1944/12/20/3432/s1, Video S1: The separation process for water-in-oil emulsion. Video S2: The separation process for oil-in-water emulsion.
